# SLC34A2 promotes neuroblastoma cell stemness via enhancement of miR‐25/Gsk3β‐mediated activation of Wnt/β‐catenin signaling

**DOI:** 10.1002/2211-5463.12594

**Published:** 2019-02-07

**Authors:** Jianlong Chen, Pengcheng Wang, Renduan Cai, Hao Peng, Chaocai Zhang, Mao Zhang

**Affiliations:** ^1^ Department of Neurosurgery Hainan General Hospital Xiuying District Haikou China

**Keywords:** cancer stem cells, Gsk3β, miR‐25, neuroblastoma, SLC34A2, stemness

## Abstract

Cancer stem cells contribute to cancer progression, but the mechanisms underlying neuroblastoma stem cell development are unclear. Here, we examined the roles of the transcription factor SLC34A2 in regulating the stemness of neuroblastoma cells. We found that SLC34A2 expression was negatively correlated with the overall survival and relapse‐free survival probability of neuroblastoma patients. Additionally, SLC34A2 expression was observed to be remarkably increased in spheroids derived from neuroblastoma cells. Knockdown of SLC34A2 attenuated the expression of stemness markers and spheroid formation capacity of neuroblastoma cell‐derived spheroids, and overexpression of SLC34A2 exerted the opposite effects in neuroblastoma cells. Mechanistically, SLC34A2 was found to directly bind to the promoter of *MIR25*, which targets glycogen synthesis kinase 3β (Gsk3β), an antagonist of Wnt signaling. Transfection of miR‐25 inhibitor or a Gsk3β overexpression plasmid attenuated the effects of SLC34A2 overexpression on the stemness of neuroblastoma cells. Our results demonstrate that miR‐25/Gsk3β‐mediated activation of Wnt signaling is responsible for SLC34A2‐induced enhancement of neuroblastoma cell stemness.

**Abbreviations**
ALDH1: aldehyde dehydrogenase 1
CSC: cancer stem cell
Gsk3β: glycogen synthesis kinase 3β
RIP: RNA immunoprecipitation
ROS: reactive oxygen species
UTR: untranslated region

Neuroblastoma is the most common primary tumor in the central nervous system; its progression is rapid and recurrence rate is high 1. Although the progress has been obtained on the treatment technology and scheme of neuroblastoma, the 5‐year survival rate of neuroblastoma patients is still < 5% 2. Cancer stem cells (CSCs) result in tumor progression 3. It is helpful for the prevention and treatment of neuroblastoma to elucidate the mechanisms underlying neuroblastoma stem cells progression.

Transcription factor SLC34A2 was first identified in 1999 and is widely expressed in various tissues and organs at different levels 4. A previous study has shown that the SLC34A2 level is upregulated in lung tissues 5, and mutations of the *SLC34A2* gene are associated with pulmonary alveolar microlithiasis 6 and lung cancer 7. SLC34A2 overexpression is an independent prognostic indicator in bladder cancer 8 and breast cancer 9. These effects indicate that SLC34A2 might be engaged in tumor progression, which was confirmed by studies showing that SLC34A2 promotes proliferation and chemoresistance in colorectal cancer through reactive oxygen species (ROS)–hypoxia‐inducible factor 1‐induced enhancer of zeste homolog 2 upregulation 10, that SLC34A2 facilitates the progression of human osteosarcoma cells through phosphatase and tensin homologue–phosphoinositide 3‐kinase–Akt signaling 11, and that SLC34A2 enhances hepatocellular carcinoma cell proliferation and invasion 12. Notably, recent research shows that SLC34A2 expression is enhanced in breast CSCs and SLC34A2 induces chemoresistance via the SLC34A2–B cell‐specific Moloney murine leukemia virus integration site 1–multidrug resistance‐associated protein 5 axis 13. However, the roles of SLC34A2 in neuroblastoma progression are still unclear.

Wnt signaling has been confirmed to be closely correlated with CSC progression 14, 15. Glycogen synthesis kinase 3β (Gsk3β), a multi‐functional serine/threonine protein kinase, could promote the phosphorylation of β‐catenin so that it can be degraded by proteasomes and subsequently inactivate Wnt signaling 16. Previous studies have shown that Gsk3β could suppress stem‐cell‐like properties and tumor growth of osteosarcoma, and induce G0/G1 arrest and apoptosis in menstrual blood‐derived endometrial stem cells through inactivating Wnt signaling 17, 18. A previous study has shown that miR‐25 could promote gastric cancer stem‐like cell progression via directly targeting Gsk3β 19. Bioinformatics analysis showed that miR‐25 is a potential target of SLC34A2 and SLC34A2 expression was negatively correlated with the survival rate of neuroblastoma patients. Notably, SLC34A2 expression was remarkably decreased in neuroblastoma cell spheroids relative to parental cells, while miR‐25 exhibited an opposite effect. Thus, we assumed that SLC34A2 might promote the stemness of neuroblastoma cells through miR‐25/Gsk3β‐mediated activation of Wnt signaling. Further ChIP and luciferase reporter assays combined with *in vitro* experiments confirmed our speculation.

## Materials and methods

### Online analysis tools

The R2 genomics analysis and visualization platform (https://hgserver1.amc.nl/cgi‐bin/r2/main.cgi) was used to analyze the correlation between SLC34A2 expression and neuroblastoma patients’ survival rate, in which Kaplan–Meier analysis by gene expression was conducted. Three represented datasets including different numbers of neuroblastoma patients were chosen for analysis: (a) Tumor Neuroblastoma public – Versteeg – 88 including 88 samples; (b) Tumor Neuroblastoma public – Kocak – 649 including 649 samples; and (c) Tumor Neuroblastoma public – SEQC – 498 including 498 samples. JASPAR^2018^ (http://jaspar.genereg.net) was used to predict the transcription factors that could bind to the promoter of MIR25.

### Cell culture

Human neuroblastoma cell line SH‐SY5Y was purchased from ATCC (Manassas, VA, USA). SH‐SY5Y cells were cultured in DMEM/F12 (1 : 1) medium (Thermo Fisher Scientific, Waltham, MA, USA) containing 2 mm l‐glutamine and 10% FBS (Thermo Fisher Scientific) under a humidified atmosphere with 5% CO_2_ at 37 °C.

### Lentivirus package

MiR‐25 overexpression and knockdown, SLC34A2 knockdown and overexpression, and Gsk3β overexpression vectors were constructed by GenePharma (Shanghai, China) and denoted as Lenti‐25, Lenti‐25‐knockdown, Lenti‐SLC34A2‐knockdown, Lenti‐SLC34A2 and Lenti‐Gsk3β, respectively. *SLC34A2* and *Gsk3β* coding sequences were inserted into pLVX‐ZsGreen vector (Addgene, Watertown, MA, USA); SLC34A2 and Gsk3β shRNA sequences were inserted into pLKO.1‐Puro vector (Addgene). Lentivirus was packaged by GenePharma.

### Quantitative real‐time PCR

Total RNA was extracted from cells using TRIzol reagent (Thermo Fisher Scientific) following the manufacturer's recommendation. Then cDNA for mRNAs was reversely synthesized using SuperScript™ First‐Strand Synthesis System for RT‐PCR (Invitrogen™, Carlsbad, CA, USA) according to the standard procedure. cDNA for miRNAs was reversely synthesized using One Step miRNA RT kit (cat. no. D1801; HaiGene, Harbin, China) and quantitative real‐time PCR (qRT‐PCR) was performed on the StepOnePlus PCR system with TransStart Green qPCR SuperMix (Transgen Biotech, Beijing, China). *GAPDH* served as an internal reference. The relative expression level of transcripts was calculated using $2^{-\Delta \Delta C_{t}}$ method.

### RNA immunoprecipitation with Ago2 assays

For the detailed procedure, refer to the previous study 20. Cells were lysed with 25 mm Tris/HCl buffer (pH 7.5) and 100 U·mL^−1^ RNase inhibitor (Sigma‐Aldrich, St. Louis, MO, USA), and then incubated with protein A Sepharose beads precoated with 3 μg anti‐Ago2 antibody or control rabbit IgG for 1.5 h at 4 °C. The RNA–protein complexes were pulled down by protein A/G agarose beads and RNA was extracted with TRIzol, followed by detecting the miR‐25 level with qRT‐PCR.

### Western blot

The detailed procedure was outlined in the previous study 21. Briefly, cells were lysed using Lysis Buffer (KeyGEN BioTECH, Nanjing, China). Protein concentration was measured using a BCA Protein Assay Kit (KeyGEN BioTECH). Twenty micrograms of protein extract was separated by 10% SDS/PAGE, then transferred to nitrocellulose membrane (Promega, Madison, WI, USA) and incubated with the primary antibodies against SLC34A2 (cat. no. 21295‐1‐AP), Nanog (cat. no. 14295‐1‐AP), aldehyde dehydrogenase 1 (ALDH1) A1 (cat. no. 15910‐1‐AP), and β‐actin (cat. no. 60008‐1‐Ig) were purchased from Proteintech (Wuhan, Hubei, P.R.C). Primary antibodies against Gsk3β (ab93926), Wnt3a (ab28472) and β‐catenin (ab32572) were purchased from Abcam (Cambridge, MA, USA). The secondary antibodies (cat. no. KGAA35 and cat. no. KGAA37) were horseradish peroxidase‐conjugated and purchased from KeyGEN BioTECH. New Super ECL (cat. no. KGP1127; KeyGEN BioTECH) was used to develop image in Tanon 5200 machine (Tanon, Shanghai, China).

### Chromatin immunoprecipitation

ChIP analysis was carried out using the SimpleChIP^®^ Enzymatic Chromatin IP Kit (agarose beads) (cat. no. 9002, Cell Signaling Technology, Danvers, MA, USA) following the manufacturer's protocols. ChIP‐grade primary antibody against SLC34A2 (cat. no. 66445) was purchased from Cell Signaling Technology. The primer sequences for the *MIR25* promoter containing the SLC34A2 binding site are as follows: forward, 5′‐TGCTCCCAGGCATTCTGGATGATAA‐3′; reverse, 5′‐TTATCATCCAGAATGCCT GGGAGCA‐3′.

### Luciferase reporter analysis

The sequences of the *MIR25* (ID: 407014; NCBI Reference Sequence: NC_000007.14) promoter, which includes the −1872 to 0 bp sequence, were inserted into a pGL3‐promoter plasmid, denoted pGL3‐miR‐25. The forward primer is 5′‐CCATGGTTCTCAGGGAAATGGTGGG‐3′; the reverse primer is 5′‐CCCACCATTTCCCTGAGAACCATGG‐3′. The sequences of the *Gsk3β* 3′‐untranslated region (UTR) were inserted into pMIR‐Report, denoted Luc‐Gsk3β‐3′UTR. The forward primer is 5′‐CTACACTTTTTCCTTTCAGAGGGGC‐3′; the reverse primer is 5′‐GCCCCTCTGAAAGGAAAAAGTGTAG‐3′. The sequences of the *MIR25* promoter with mutated binding site of SLC34A2, and the sequences of *Gsk3β* 3′‐UTR with mutated binding sites of miR‐25 were obtained using Fast Mutagenesis Kit V2 (Vazamy, Nanjing, China) according to the manufacturer's instructions, and named pGL3‐miR‐25‐mut and Luc‐Gsk3β‐3′UTR‐mut, respectively. For confirming the miR‐25 targeting on *Gsk3β*, Luc‐Gsk3β‐3′UTR or Luc‐Gsk3β‐3′UTR‐mut was co‐transfected with miR‐25 mimics or negative control, and β‐gal plasmid into glioma cells using Lipofectamine 2000 Reagent (Waltham, MA, USA) following the manufacturer's recommendation. For confirming the interaction between SLC34A2 and the *MIR25* promoter, pGL3‐miR‐25 or pGL3‐miR‐25‐mut was co‐transfected with Lenti‐SLC34A2 infection; 72 h later, cells were lysed with Reporter lysis buffer (cat. no. E397A; Promega Corp.) and luciferase activity was measured with VivoGlo Luciferin kit (cat. no. P1041; Promega Corp.) using a luminometer (Thermo Fisher Scientific) and normalized to β‐gal activity.

### Spheroid formation assay

This process was outlined in the previous study 22. Neuroblastoma cell spheroid formation was performed under anchorage‐independent conditions in methylcellulose (Sigma‐Aldrich). Briefly, neuroblastoma cells with different treatment were digested with Trypsin/EDTA (Sigma‐Aldrich), and then cultured in DMEM/F12 medium supplemented with B27 (20 ng·mL^−1^) and epidermal growth factor (10 ng·mL^−1^) in non‐adherent 24‐well plates at 500 cells/well. After 8 days, spheres > 50 μm were counted. This experiment was performed in triplicate and repeated at least three times independently. Additionally, for experiments on spheroids, spheroids were trypsinized, re‐seeded into plates and subsequently used in the experiments.

### Statistical analysis

All results are denoted as mean ± SD and analyzed using prism (version X; GraphPad Software, La Jolla, CA, USA). Student's *t* test was used to assess the differences between groups. *P* < 0.05 or less was considered statistically significant.

## Results

### SLC34A2 expression is negatively correlated with the overall survival and relapse‐free survival of neuroblastoma patients

**Figure 1:**
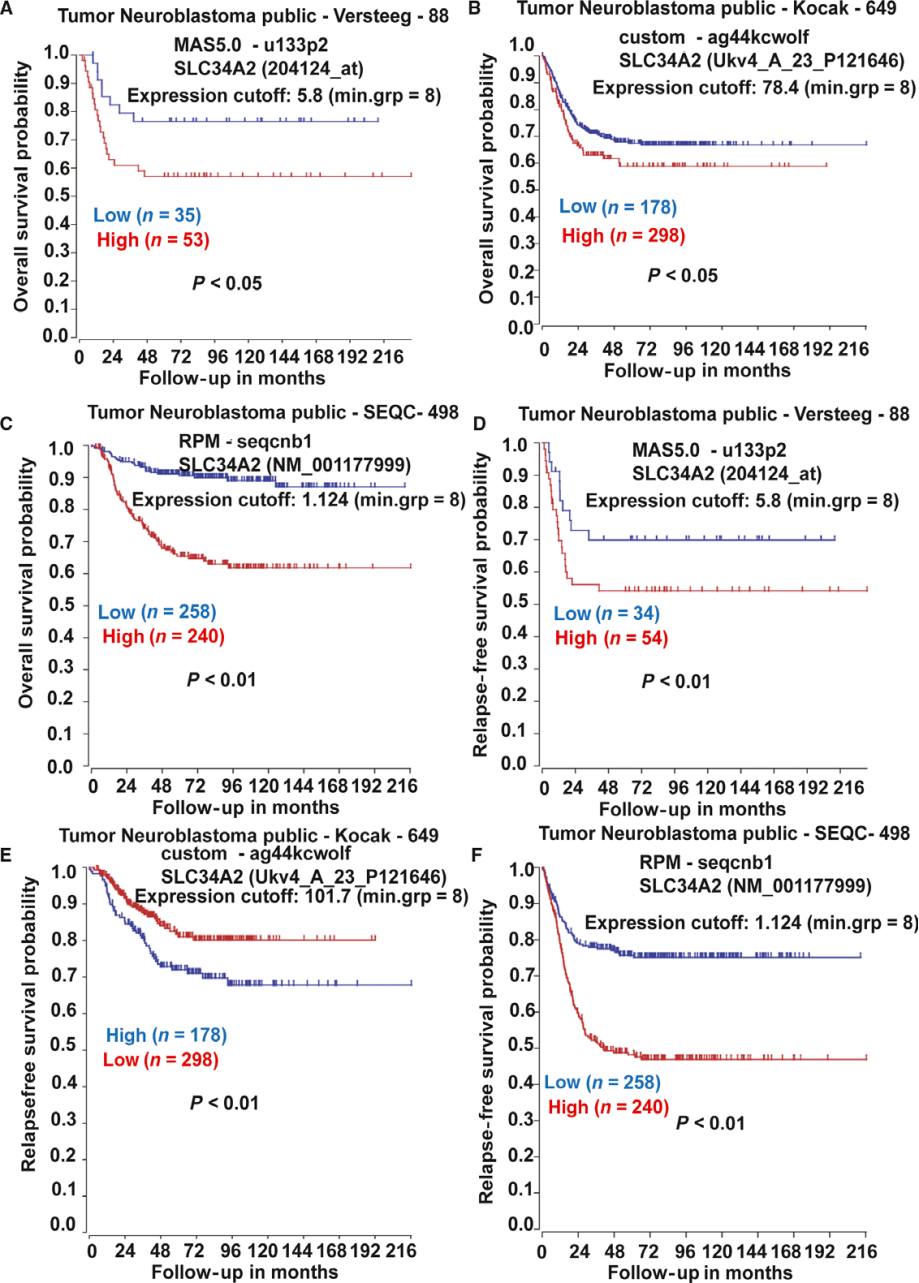
SLC34A2 expression is negatively correlated with the overall survival and event‐free survival of neuroblastoma patients. (A–F) KM‐Plotter analysis showed the correlation between SLC34A2 expression and overall survival (A–C) and relapse‐free survival (D–F) of neuroblastoma patients.

We firstly evaluated the correlation between SLC34A2 expression and the survival of neuroblastoma patients. The R2 genomics analysis and visualization platform (https://hgserver1.amc.nl/cgi-bin/r2/main.cgi) was used based on three represented dataset including different numbers of neuroblastoma patients. As shown in Fig. 1A–F, SLC34A2 expression was negatively correlated with the overall survival (Fig. 1A–C) and relapse‐free survival (Fig. 1D–F) of neuroblastoma patients. These results indicate that SLC34A2 might be engaged in neuroblastoma progression.

### SLC34A2 promotes the stemness of neuroblastoma cells

**Figure 2:**
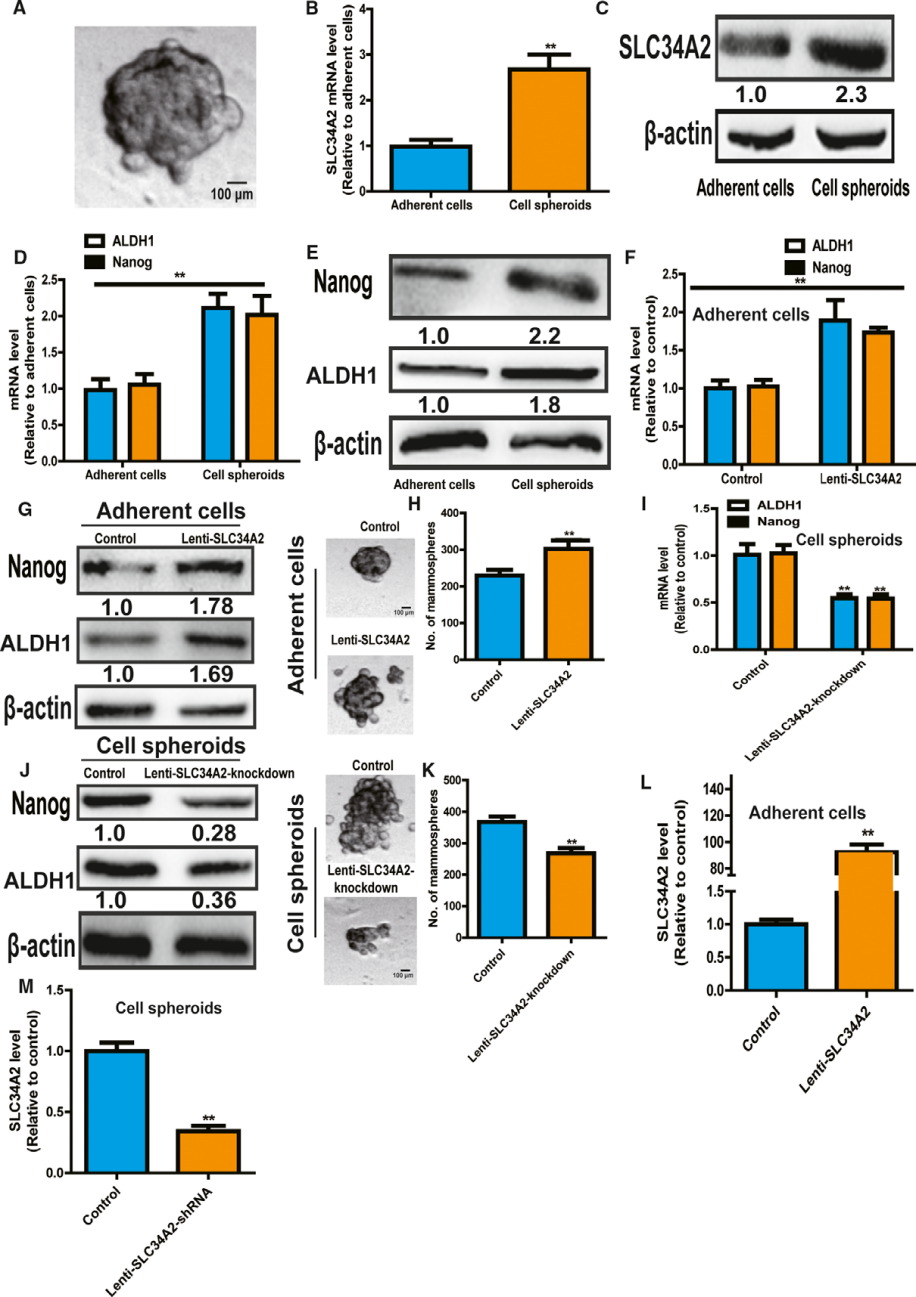
SLC34A2 promotes the stemness of neuroblastoma cells. (A) Representative image of spheroids formed by SH‐SY5Y cells. Scale bar, 100 μm. (B,C) SLC34A2 expression was detected in SH‐SY5Y cells and spheroids. (D,E) Expression of stemness markers (ALDH1 and Nanog) was examined in SH‐SY5Y cells and spheroids. (F,G) Expression of stemness markers was determined in SH‐SY5Y cells with or without SLC34A2 overexpression. (H) The capacity of spheroid formation was evaluated in cells depicted in (F). Scale bar, 100 μm. (I,J) Expression of stemness markers was measured in SH‐SY5Y cell spheroids with or without SLC34A2 knockdown. (K) The capacity of spheroid formation was detected in spheroids described in (I). (L,M) The overexpression and knockdown efficiency of SLC34A2 was confirmed in SH‐SY5Y cells and spheroids, respectively. The difference was assayed using one‐way ANOVA with the Tukey–Kramer *post hoc* test. Data are presented as mean ± SD; *n* ≥ 3, **P < 0.01 *vs* control.

Since CSCs contribute to tumor progression, we explored whether SLC34A2 regulates the stemness of neuroblastoma cells. First, we collected spheroids formed by neuroblastoma SH‐SY5Y cells (Fig. 2A). qRT‐PCR and western blot analysis indicated that SLC34A2 expression was significantly increased in cell spheroids formed by SH‐SY5Y cells (Fig. 2B,C). Notably, expression of stemness markers (ALDH1 and Nanog) displayed a higher level in spheroids relative to parental SH‐SY5Y cells (Fig. 2D,E). These results suggest that SLC34A2 might be involved in regulating the stemness of SH‐SY5Y cells. As expected, SLC34A2 overexpression significantly increased the stemness marker expression and the spheroids formation capacity of SH‐SY5Y cells (Fig. 2F–H). Consistently, SLC34A2 knockdown in spheroids formed by SH‐SY5Y cells exerted opposite effects (Fig. 2I–K). The knockdown and overexpression efficiency of SLC34A2 was confirmed by qRT‐PCR (Fig. 2L,M). Our results demonstrate that SLC34A2 promotes the stemness of neuroblastoma cells.

### SLC34A2 directly binds to the promoter of *MIR25* and increases its expression in neuroblastoma cells and spheroids

**Figure 3:**
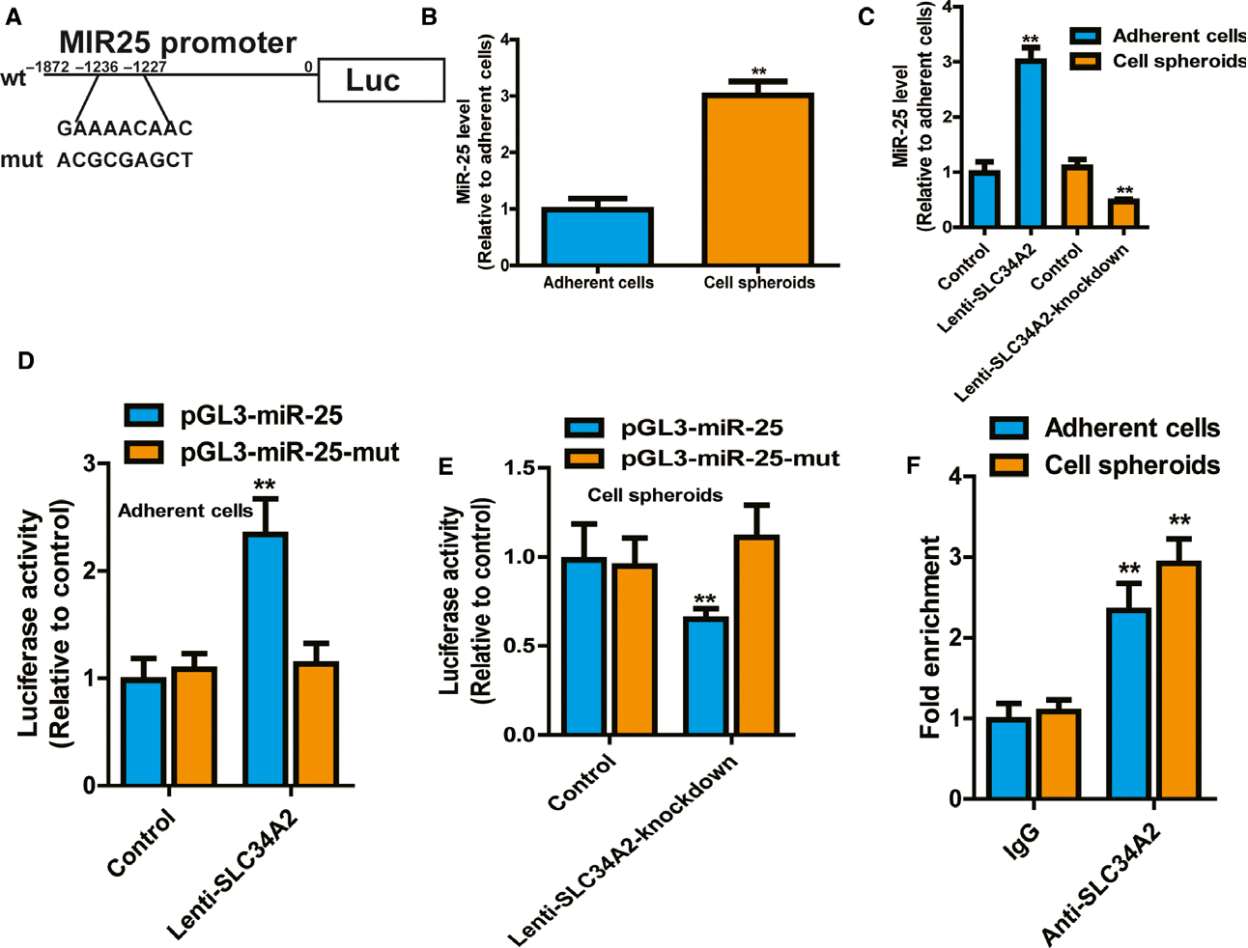
SLC34A2 directly binds to the promoter of *MIR25* and increases its expression in neuroblastoma cells and spheroids. (A) Diagram of *MIR25* promoter containing SLC34A2 binding site and corresponding mutant site. (B) MiR‐25 level was examined in SH‐SY5Y cells and spheroids. (C) MiR‐25 level was determined in SH‐SY5Y cells with SLC34A2 overexpression or spheroids with SLC34A2 knockdown. (D) The luciferase activity of pGL3‐miR‐25 and pGL3‐miR‐25‐mut was measured in SH‐SY5Y cells with or without SLC34A2 overexpression. (E) The luciferase activity of pGL3‐miR‐25 and pGL3‐miR‐25‐mut was measured in SH‐SY5Y cells with or without SLC34A2 knockdown. (F) ChIP assay was performed in SH‐SY5Y cells with anti‐SLC34A or IgG, followed by determination of miR‐25 level. The difference was assayed using one‐way ANOVA with the Tukey–Kramer *post hoc* test. Data are presented as mean ± SD; *n* ≥ 3, **P < 0.01 *vs* control.

Previous study has shown that SLC34A2 could target the *MIR25* promoter to promote gastric cancer progression (Fig. 3A) 19; we wondered whether the SLC34A2–miR‐25 axis exists in neuroblastoma cells. Notably, the miR‐25 level exhibited a similar effect in SH‐SY5Y cells and spheroids, characterized as an increase in spheroids derived from SH‐SY5Y cells (Fig. 3B). qRT‐PCR showed that SLC34A2 overexpression promoted miR‐25 expression in SH‐SY5Y cells, and knockdown of SLC34A2 decreased miR‐25 expression in spheroids derived from SH‐SY5Y cells (Fig. 3C). Furthermore, luciferase reporter analysis indicated that SLC34A2 overexpression increased the luciferase activity of pGL3‐miR‐25 in SH‐SY5Y cells, while knockdown of SLC34A2 decreased pGL3‐miR‐25, but had no effect on pGL3‐miR‐25‐mut (Fig. 3D,E). Additionally, we performed a ChIP assay in SH‐SY5Y cells and spheroids with an antibody against SLC34A2. Our results indicated that *MIR25* promoter sequences with SLC34A2 potential binding site were enriched in DNA pulled down by anti‐SLC34A2 in SH‐SY5Y cells and spheroids (Fig. 3F).

### MiR‐25 directly binds to Gsk3β 3′UTR and thus activates Wnt signaling

**Figure 4:**
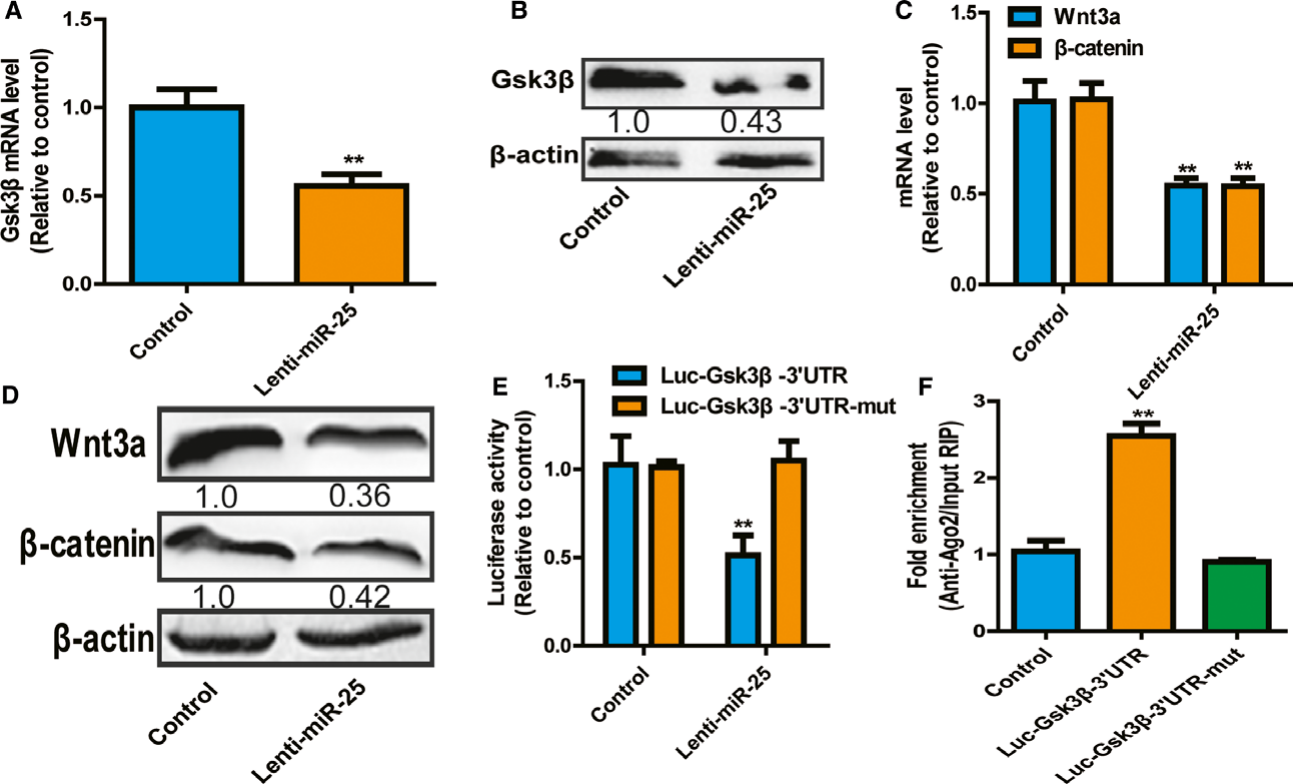
MiR‐25 directly binds to *Gsk3β* 3′UTR and thus activates Wnt signaling. (A,B) Gsk3β mRNA (A) and protein (B) levels were determined in SH‐SY5Y cells with or without miR‐25 overexpression. (C,D) Expression of Wnt3a and β‐catenin was examined in SH‐SY5Y cells with or without miR‐25 overexpression. (E) Luciferase reporter assay was performed in SH‐SY5Y cells with or without miR‐25 overexpression. (F) RIP analysis was carried out in SH‐SY5Y cells with or without Luc‐Gsk3β‐3′UTR or Luc‐Gsk3β‐3′UTR‐mut overexpression. The difference was assayed using one‐way ANOVA with the Tukey–Kramer *post hoc* test. Data were presented as mean ± SD; *n* ≥ 3, **P < 0.01 *vs* control.

We then investigated the downstream effectors of miR‐25. Bioinformatics analysis (targetscan 6.2, http://www.targetscan.org/vert_72/) indicated that Gsk3β, an antagonist of Wnt signaling contributing to stemness of cancer cells, was a potential target of miR‐25. Firstly, qRT‐PCR and western blot analysis showed that miR‐25 overexpression decreased Gsk3β expression in SH‐SY5Y cells (Fig. 4A,B). Notably, the downstream Wnt signaling was activated, evidenced by the increase of Wnt3a and β‐catenin expression (Fig. 4C,D). Additionally, a luciferase reporter assay indicated that miR‐25 overexpression reduced the luciferase activity of Luc‐Gsk3β‐3′UTR, but had no effect on the luciferase activity of Luc‐Gsk3β‐3′UTR‐mut in SH‐SY5Y cells (Fig. 4E). Furthermore, RNA immunoprecipitation (RIP) analysis was performed to pull down endogenous miRNAs associated with Ago2 in Luc‐Gsk3β‐3′UTR‐overexpressing or Luc‐Gsk3β‐3′UTR‐mut cells. The precipitated miRNAs were subjected to qRT‐PCR analysis and the results showed that miR‐25 was enriched in RNAs retrieved from Luc‐Gsk3β‐3′UTR‐overexpressing cells, but not in Luc‐Gsk3β‐3′UTR‐mut overexpressing cells (Fig. 4F). These results support that SLC34A2 is the bona fide target of miR‐25 in SH‐SY5Y cells.

### SLC34A2 attenuates the stemness of neuroblastoma cells through the miR‐25–Gsk3β axis

**Figure 5:**
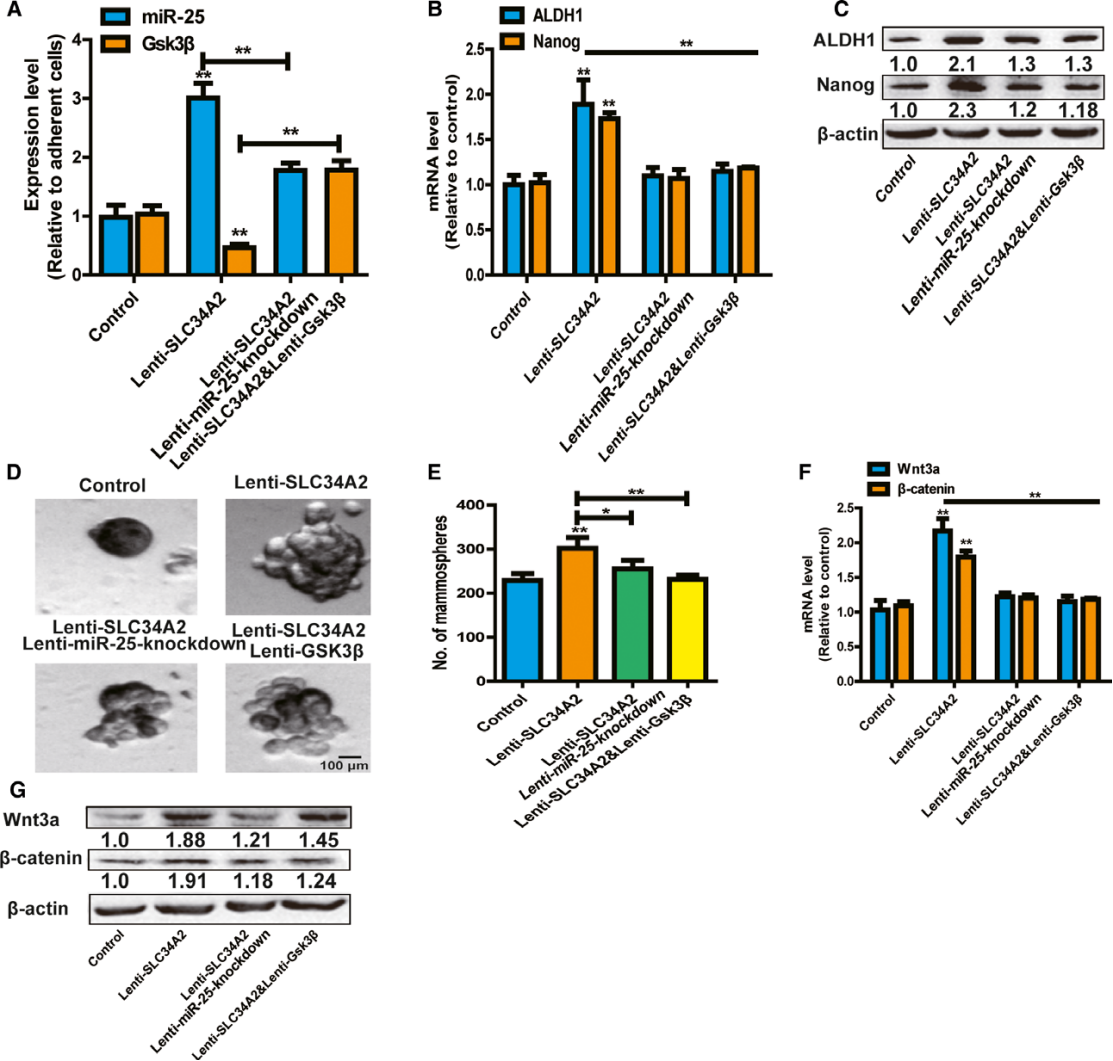
SLC34A2 attenuates the stemness of neuroblastoma cells through miR‐25–Gsk3β axis. (A) MiR‐25 and Gsk3β expression was detected in SH‐SY5Y cells with SLC34A2 overexpression plus miR‐25 knockdown or Gsk3β overexpression. (B,C) Expressions of stemness markers were measured in cells depicted in (A). (D,E) The capacity of spheroid formation was determined in cells described in (A) by detecting the spheroid size (D) and number (E). Scale bar, 100 μm. (F,G) Expression of Wnt3a and β‐catenin was examined in cells depicted in (A). The difference was assayed using one‐way ANOVA with the Tukey–Kramer *post hoc* test. Data are presented as mean ± SD; *n* ≥ 3, *P < 0.05, **P < 0.01 *vs* control.

Finally, we investigated whether SLC34A2 attenuated the stemness of SH‐SY5Y cells through miR‐25–Gsk3β signaling. MiR‐25 was knockdown or Gsk3β was overexpressed in SH‐SY‐5Y cells with SLC34A2 overexpression (Fig. 5A). As expected, knockdown of miR‐25 or Gsk3β overexpression attenuated the promoting effects of SLC34A2 overexpression on the expression of stemness markers (Fig. 5B,C). Additionally, the increase of spheroid formation capacity mediated by SLC34A2 overexpression was partially abrogated by miR‐25 knockdown or Gsk3β overexpression in SH‐SY5Y cells (Fig. 5D,E). Notably, SLC34A2 overexpression indeed activated Wnt signaling, characterized as the increase of Wnt3a and β‐catenin expression; this effect was partially rescued by miR‐25 knockdown or Gsk3β overexpression (Fig. 5F,G). Therefore, these results suggest that SLC34A2 facilitates the stemness of SH‐SY5Y cells at least through the miR‐25–Gsk3β axis.

## Discussion

Previous studies have shown that *ROS–SLC34A2* fusion genes could promote tumor cells proliferation in glioma and non‐small cell lung cancer 23, 24; this effect was regarded as being associated with the proliferation and invasion abilities of CSCs 13. Further studies have indicated that SLC34A2 knockdown attenuates the proliferation of lung CSCs 25, and SLC34A2 overexpression facilitates the stemness of and confers chemoresistance on breast cancer cells 13, 26. However, the roles of SLC34A2 in neuroblastoma progression remain unclear.

Since CSCs contribute to tumor progression, here we focused on the role of SLC34A2 in regulating the stemness of neuroblastoma cells. Since there are no specific markers for neuroblastoma stem cells, we collected the spheroids, which had been confirmed as being enriched in CSCs 20, 27. Notably, we found that SLC34A2 expression was significantly increased in spheroids formed by SH‐SY5Y cells, which prompted us to explore the roles of SLC34A2 in the stemness of neuroblastoma cells. As expected, we revealed that SLC34A2 positively regulated the stemness of neuroblastoma cells through a spheroid formation assay. To the best of our knowledge, this is the first study showing a role for SLC34A2 in neuroblastoma cell stemness. However, we must admit that the conclusion should be validated by *in vivo* experiment, and future works could focus on the role of SLC34A2 in regulating other functions of neuroblastoma cells, such as cell migration and invasion.

Mechanistically, we found that SLC34A2 could directly bind to the promoter of *MIR25* and thus upregulate its level, which is consistent with the previous study showing that SLC34A could regulate miR‐25 activity to affect gastric cancer progression 19. As miRNAs exert their function through their downstream targets, we searched for potential targets of miR‐25; Gsk3β attracted our attention as an antagonist of Wnt signaling which promotes CSC progression. We further confirmed our prediction through RIP and luciferase reporter assays. Finally, we demonstrated that SLC34A2‐mediated effects on the stemness of neuroblastoma cells were at least through the miR‐25–Gsk3β axis, establishing the SLC34A2–miR‐25–Gsk3β regulatory axis in neuroblastoma cells. However, we cannot exclude that there are other pathways involved in SLC34A2‐mediated effects in neuroblastoma cell stemness.

In conclusion, our results indicate that SLC34A2 could directly bind to the promoter of *MIR25* and thus facilitate miR‐25–Gsk3β axis‐mediated activation of Wnt signaling, which is responsible for the SLC34A2‐mediated effects on the stemness of neuroblastoma cells. Importantly, SLC34A2 expression is negatively correlated with the overall survival of neuroblastoma patients. This work provides evidence confirming that SLC34A2 could be a novel candidate for neuroblastoma treatment or prognosis.

## Conflict of interest

The authors declare no conflict of interest.

## Author contributions

JC and PW conceived and designed the project; JC, RC and HP acquired the data; CZ and MZ analyzed and interpreted the data; and JC and PW wrote the paper.
